# Treatment of aneurysmal artery with PED: A case report

**DOI:** 10.1097/MD.0000000000036377

**Published:** 2023-12-01

**Authors:** Da Li, Yunxia Jiang, Chengjun Zhuge, An Wu

**Affiliations:** a The Second School of Clinical Medicine, Zhejiang Chinese Medical University, Hangzhou, People’s Republic of China; b The Second School of Clinical Medicine, Zhejiang Chinese Medical University, Hangzhou, People’s Republic of China; c The Second School of Clinical Medicine, Zhejiang Chinese Medical University, Hangzhou, People’s Republic of China; d Department of Neurosurgery, The Quzhou Affiliated Hospital of Wenzhou Medical University, Quzhou People’s Hospital, Quzhou, People’s Republic of China.

**Keywords:** case report, flow diverter stent and embolization, parent artery, saccular aneurysm, wall shear stress

## Abstract

**Rationale:**

Pipeline embolization devices are a new treatment for intracranial aneurysms, especially irregular, giant aneurysms.

**Patient concerns:**

A 48-year-old female patient presented with a headache in the frontal part for 3 weeks.

**Diagnoses:**

Cerebral computed tomographic angiography and magnetic resonance angiography showed a saccular aneurysm in the right internal carotid artery that wrapped the parent artery. Digital subtraction angiography provided images with 1 large saccular internal carotid aneurysm.

**Interventions:**

The patient was treated by flow diverter stent and coil embolization and dual antiplatelet therapy with aspirin and ticagrelor in the ICU and was discharged after 10 days without complications.

**Outcomes:**

One year after interventional therapy, repeated digital subtraction angiography showed no recurrence of aneurysm and embolization well, and the patient reported improvement in neurological symptoms.

**Lessons:**

Aneurysmal parent artery is a rare phenomenon. The combination of flow diverter stents and coil embolization to treat cases with large saccular aneurysms has important clinical significance and this may provide a reference for clinical treatment of aneurysmal parent artery.

## 1. Introduction

Intracranial aneurysms (IAs) are abnormal bulges on the wall of the intracranial artery, and they occur in approximately 3% to 5% of the population.^[1]^ According to statistics, internal carotid artery aneurysm is the most common type, accounting for approximately 38% of cases.^[2]^ The parent artery is significant for the formation of aneurysms. For example, it is easier to form aneurysms at the bifurcation of the anterior communicating artery, and distortion of the parent artery is related to the presence of aneurysms in any segment of the brain.^[3,4]^ Aneurysmal parent artery is a rare phenomenon, and we present a case of using pipeline embolization device (PED) for treatment.

## 2. Case report

### 2.1. History

A 48-year-old woman had a headache in the frontal region for 3 weeks. The pain was continuous, and she had no other neurological symptoms. During the illness, she experienced headache without treatment and attention. However, the symptoms aggravated gradually after 6 days, and she underwent magnetic resonance angiography at a local inspection institution, which showed a right middle cerebral artery M1 segment aneurysm. The patient was transferred to our institution for further treatment.

**Figure 1.**
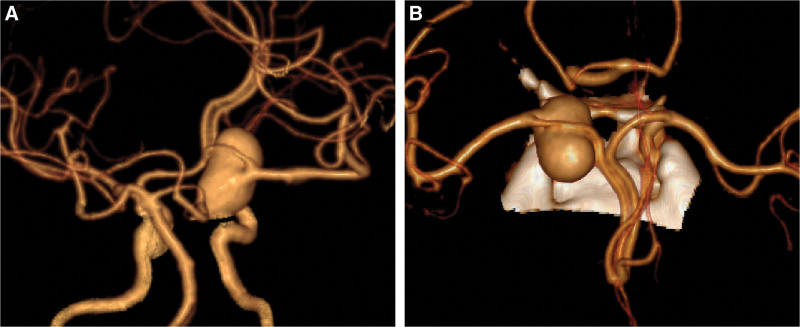
The aneurysm that was characterized by no aneurysm neck and complete aneurysmization on the ICA C7 segment.

**Figure 2.**
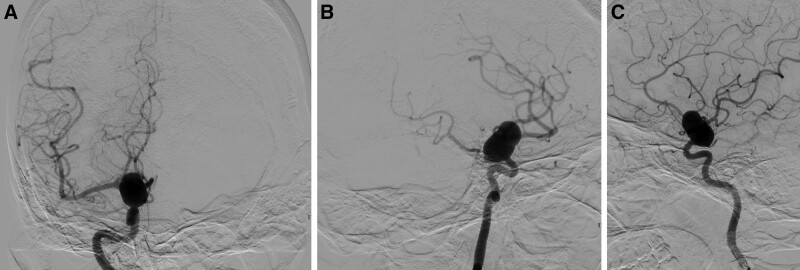
Right internal carotid arteriography: a large aneurysm was located the C7 bifurcation, and wrapping of the MCA, COA and PCA, and other branches was observed. COA = cerebral ophthalmic artery, PCA = posterior communicating artery.

At the time of the patient’s arrival, she had no new neurological symptoms except headache, and her Glasgow coma scale score was 15. We performed a computed tomographic angiography scan of the brain artery at the time of admission, which revealed a medium-sized saccular aneurysm formed on the right internal carotid artery. However, it completely wrapped the C7 bifurcation artery (Fig. 1) and showed a 12*22 mm aneurysm with a smooth wall, irregular shape and no abnormal bulge by digital subtraction angiography (Fig. 2).

The patient had mild nerve injury and was in a stable condition, but the risk of aneurysm rupture was high. Three days after the completion of intracranial angiography, the patient signed the informed consent document, and our institution decided to perform embolization of the aneurysm with a stent.

### 2.2. Operation

**Figure 3.**
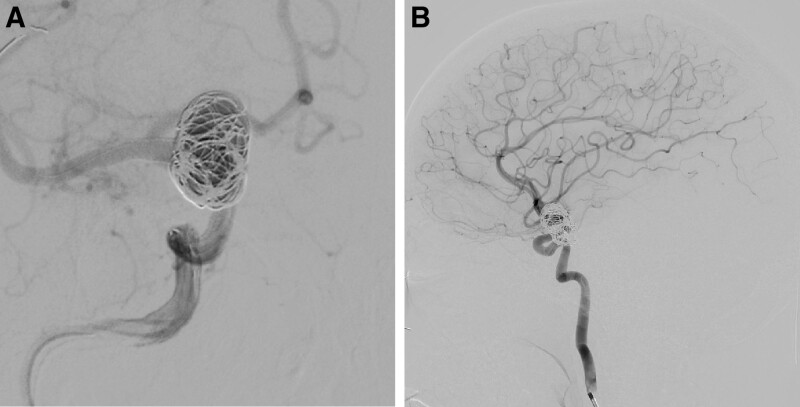
(A and B) DSA images during the operation and postoperatively. (A) Flow diverter stent and coil embolization were released well in the ICA and aneurysm (B)1 year follow-up after operation. DSA = digital subtraction angiography.

The patient underwent heparinization, and the sheath was placed from the right femoral artery. Angiography showed the aneurysm, and 3D angiography confirmed no other abnormalities. The guiding catheter was placed in the C2 segment of the right internal carotid artery, and the blood flow diverter PED stent was placed immediately after the stent was placed. When the stent was released, angiography was performed again, showing that the blood flow was unobstructed and that the stent had adhered well. Finally, the aneurysm was filled with coils. Angiography showed that the stent was well-open, the intracranial vessels were improved, and the Willis ring was compensated. Postoperative angiography confirmed that the aneurysm occlusion was good, and the internal carotid artery and its branches remained unobstructed (Fig. 3).

The patient was monitored postoperatively in the intensive care unit. Neurologically, she continued to improve and made an excellent recovery with no new focal deficits.

## 3. Discussion

The first appearance of IA was described by the Italian anatomist Morgani in 1761; it was an unruptured aneurysm on the carotid artery, and people began to pay attention to changes in intracranial blood vessels.^[5]^ According to the International Study of Unruptured Intracranial Aneurysms classification, the aneurysms in this report were classified as large (small < 10 mm; large < 10–25 mm; giant > 25 mm).^[6]^ SAH caused by rupture is the most serious complication of IAs and has high disability and mortality rates.^[7,8]^ However, nonwounded aneurysm rupture still accounts for 0.95% of cases per year,^[9]^ and the mortality rate is close to 50%.^[10]^

Research on IAs is still in progress, and wall shear stress (WSS) may be one of the reasons for the formation and rupture of IAs. Shakur et al^[11]^ found that the increase in WSS caused by ICA occlusion is a risk factor for IA growth. According to the study of Leemans et al,^[12]^ the width of the aneurysm is considered the most significant part of the growth during the growth of aneurysms. Interestingly, Cornelissen et al^[13]^ found a significant decrease in this value after 4 years of aneurysm follow-up,^[13]^ and most studies also confirmed that the decrease in this value may be a risk factor for aneurysm rupture.^[14,15]^ The reason is that hemodynamic may promote the inflammatory response in the blood vessels and cause the growth or rupture of aneurysms.^[16]^ Zhou et al^[17]^ found that these inflammatory reactions promoted the production of metalloproteinases and further thinned the arterial wall. Wang et al^[18]^ also noted that the ability of endothelial cells to withstand haemodynamic pressure is reduced by WSS, which promotes aneurysm rupture.

The PED technology used in this case tends to be mature. By changing the direction of blood flow, the endothelial cells in the aneurysm have a favorable environment of growth, which is conducive to the occlusion of the tumor and vascular reconstruction.^[19]^ The postoperative embolization rates of patients similar to this case were 85%, 95%, and 100%.^[20]^ In fact, this patient recovered well after the operation, and the embolization rate was approximately 95% after 1 year of follow-up. In this report, the patient’s neurological symptoms were relatively singular, and after CFD analysis, it was found that the preoperative WSS distribution of the patients was uneven. From near to far, the WSS at the parent artery was higher, and the internal distribution of the tumor changed greatly. The farther away from the parent artery, the lower the WSS. This report also reveals that WSS is related to the formation or rupture of aneurysms. Changing the tumor of intravascular WSS has very important clinical significance for the growth and rupture of aneurysms.

## Acknowledgements

The authors thank all staffs in Department of Neurosurgery, People’s Hospital of Quzhou (Quzhou China) for their technical support.

## Author contributions

**Conceptualization:** Da Li.

**Data curation:** Da Li.

**Funding acquisition:** An Wu.

**Software:** Yunxia Jiang.

**Validation:** An Wu.

**Visualization:** Yunxia Jiang.

**Writing – original draft:** Da Li.

**Writing – review & editing:** Da Li, Chengjun Zhuge.
